# A Reconsideration of Acute Beryllium Disease

**DOI:** 10.1289/ehp.0800455

**Published:** 2009-04-28

**Authors:** Kristin J. Cummings, Aleksandr B. Stefaniak, M. Abbas Virji, Kathleen Kreiss

**Affiliations:** Division of Respiratory Disease Studies, National Institute for Occupational Safety and Health, Centers for Disease Control and Prevention, Morgantown, West Virginia, USA

**Keywords:** acute, beryllium, beryllium disease, granuloma, hypersensitivity, immune sensitization, pneumonitis

## Abstract

**Context:**

Although chronic beryllium disease (CBD) is clearly an immune-mediated granulomatous reaction to beryllium, acute beryllium disease (ABD) is commonly considered an irritative chemical phenomenon related to high exposures. Given reported new cases of ABD and projected increased demand for beryllium, we aimed to reevaluate the patho physiologic associations between ABD and CBD using two cases identified from a survey of beryllium production facility workers.

**Case Presentation:**

Within weeks after exposure to beryllium fluoride began, two workers had systemic illness characterized by dermal and respiratory symptoms and precipitous declines in pulmonary function. Symptoms and pulmonary function abnormalities improved with cessation of exposure and, in one worker, recurred with repeat exposure. Bronchoalveolar lavage fluid analyses and blood beryllium lymphocyte proliferation tests revealed lymphocytic alveolitis and cellular immune recognition of beryllium. None of the measured air samples exceeded 100 μg/m^3^, and most were < 10 μg/m^3^, lower than usually described. In both cases, lung biopsy about 18 months after acute illness revealed noncaseating granulomas. Years after first exposure, the workers left employment because of CBD.

**Discussion:**

Contrary to common understanding, these cases suggest that ABD and CBD represent a continuum of disease, and both involve hypersensitivity reactions to beryllium. Differences in disease presentation and progression are likely influenced by the solubility of the beryllium compound involved.

**Relevance to Practice:**

ABD may occur after exposures lower than the high concentrations commonly described. Prudence dictates limitation of further beryllium exposure in both ABD and CBD.

In 2004, South Korean investigators reported nine cases of a disease thought to have been eliminated decades before: acute beryllium disease (ABD) (Kim et al. 2004). The recognition of new cases, the projected growth in worldwide demand for beryllium for applications including nuclear energy production (U.S. Department of Energy 2008) and national defense (Business Wire 2005), and advances in the understanding of chronic beryllium disease (CBD) led us to reconsider the pathogenesis of ABD. The resulting reconceptualization has implications for prevention, diagnosis, and case management in the global beryllium industry.

Textbooks and review articles have stated that exposure to beryllium may result in two distinct respiratory conditions. ABD is considered to be an irritative chemical phenomenon, whereas CBD is recognized as an immune-mediated granulomatous process (Balmes 2005; Becklake and Cowie 2000; Churg and Green 1998; Williams 1988). This conceptualization began > 50 years ago, with the assertions that ABD followed a traditional exposure–response pattern and was associated with airborne beryllium concentrations > 100 μg/m^3^, whereas CBD could occur at much lower levels, indicating an immune phenomenon (Sterner and Eisenbud 1951). Although the description of ABD has remained essentially static, the understanding of CBD has evolved greatly in recent decades. It is now well established that sensitization to beryllium, as measured by the beryllium lymphocyte proliferation test (BeLPT), reflects cellular immune recognition of beryllium and confers a higher risk of subsequent development of CBD (Kreiss et al. 2007; Mroz et al. 1991; Newman et al. 2005; Sawyer et al. 2002). CBD can be detected at a subclinical stage by bronchoalveolar lavage (BAL) and biopsy (Cordeiro et al. 2007; Maier 2001). Lymphocyte predominance and abnormal BeLPT on BAL fluid analysis are findings consistent with CBD.

Acute respiratory and dermal reactions to beryllium exposure were first reported in the United States in the 1940s, observed among workers in the beryllium extraction and processing industry (DeNardi et al. 1949; Van Ordstrand et al. 1945). A relationship with exposure to soluble beryllium salts (sulfate and fluoride) and soluble forms of the oxide was noted by early investigators (Eisenbud 1982; Eisenbud et al. 1948). During that era, daily weighted average (DWA) exposures to beryllium were known to exceed 1,000 μg/m^3^ in certain operations (Eisenbud 1982).

In 1949, the U.S. Atomic Energy Commission recommended two different occupational exposure limits to their contractors: 25 μg/m^3^ as a maximum permissible peak exposure, to prevent ABD, and 2 μg/m^3^ as a DWA over a quarterly period, to prevent CBD (Eisenbud 1982). These limits were subsequently adopted in the United States by various professional organizations and the Occupational Safety and Health Administration, and regulatory bodies in many other countries also recognize the 2 μg/m^3^ limit (Eisenbud 1982, 1998). Over time, average exposures have decreased from hundreds of micrograms per cubic meter in the 1940s and 1950s to ≤ 1 μg/m^3^ in the 1980s and 1990s (National Research Council 2008).

Beryllium fluoride is intentionally formed during the production of beryllium metal. In the initial step, ammonium beryllium fluoride is heated in a fluoride furnace to drive off ammonium fluoride gas and yield beryllium fluoride (Kroschwitz and Howe-Grant 1992; White and Burke 1955). The beryllium fluoride then is transferred to an adjacent reduction furnace and reacted with magnesium to yield beryllium metal. In this article, we describe two cases of acute respiratory and dermal illness that occurred in workers involved in beryllium metal production. The extensive diagnostic evaluations that they underwent provide details on pathogenesis that were not available in earlier reports of ABD. In light of these cases and a review of the historical literature, we suggest that rather than being two distinct clinical entities, ABD and CBD represent points on a continuum of hypersensitivity reactions to beryllium.

## Methods

Cases were identified through a survey of workers at a beryllium manufacturing plant producing pure metal, oxide, and alloys (Kreiss et al. 1997). To prepare the case reports, we reviewed medical records of care provided in the plant’s medical clinic or at referral health care facilities and examined chest radiographs taken in the plant’s medical clinic. We used the plant’s air sampling data to characterize exposures that occurred before symptom onset. Air measurements included short-duration (< 30 min) breathing zone (BZ) air samples, short- or long-duration (5 min to 8 hr) general area (GA) air samples, and DWA exposures calculated by combining the GA and BZ air samples with information on typical amount of time spent at different locations and activities by job titles. From these GA or BZ sample data, we selected the highest time-weighted average (TWA) beryllium concentration on any given day from the work areas or activities associated with a worker’s job and tenure. Each case is presented with the worker’s written permission and institutional review board approval.

## Case 1

**Figure 1:**
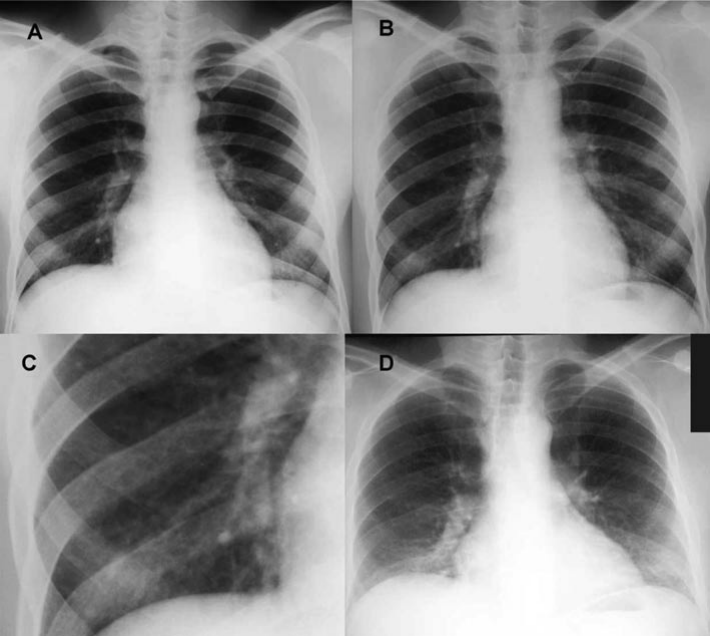
Chest radiographs of case 1. (*A*) Radiograph taken pre hire on 2 March 1979, showing normal lung fields. (*B* ) Radiograph taken during the second episode of acute work-related illness on 17 March 1981, showing a mild diffuse nodular infiltrate; (*C*) close-up of the right lower lung field from radiograph in (*B*). (*D* ) Radiograph at follow-up on 13 February 1997, showing reduced lung volumes and a bilateral interstitial infiltrate.

**Figure 2:**
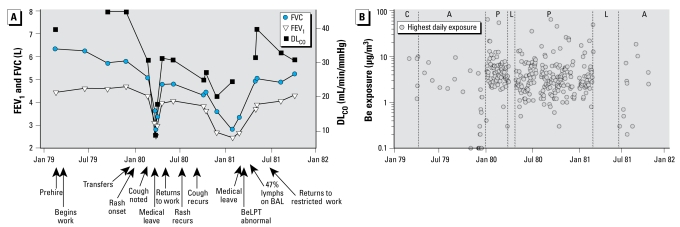
Summary of lung function and beryllium exposure of case 1. (*A*) Results of pulmonary function tests before, during, and after two episodes of acute work-related illness. (*B*) TWA airborne beryllium exposures in patient’s departments during this time period. Abbreviations: A, alloy department exposures (beryllium metal, beryllium oxide, copper); C, ceramics department exposures (beryllium oxide); L, medical leave; P, metals production department exposures (beryllium metal, beryllium oxide, beryllium fluoride, ammonium beryllium fluoride, ammonium fluoride, magnesium fluoride).

A healthy 20-year-old male nonsmoker began working at the beryllium production plant on 12 March 1979. His preemployment chest radiograph was normal (Figure 1A) and pulmonary function tests were normal, including a forced vital capacity (FVC) of 6.34 L (120% predicted) and a carbon monoxide diffusing capacity (DL_CO_) of 39.5 mL/min/mmHg (119% predicted) (Figure 2A). After time in the ceramics and alloy fabrication departments (Kent et al. 2001; Stefaniak et al. 2003, 2004), during which routine pulmonary function tests were normal, he was transferred on 31 December 1979 to the metal production department to operate the reduction furnace (Kent et al. 2001; Kroschwitz and Howe-Grant 1992; Stefaniak et al. 2003, 2004; White and Burke 1955). On 12 January 1980, he presented to the plant’s medical office complaining of a new rash on both forearms and was seen multiple times for the rash over the next month.

On 29 March 1980, the patient complained of shortness of breath, chest pain, and a non productive cough that had begun several weeks earlier. He had lost 2.7 kg over the preceding month. Pulmonary function testing demonstrated a substantial fall in FVC, to 3.41 L (64% predicted), and a decrease in DL_CO_, to 8.9 mL/min/mmHg (27% predicted). The chest radiograph was normal. On 1 April 1980, a company physician noted “scattered rales” on chest examination and restricted the patient from further work.

While on work restriction, the patient was treated with antibiotics and antitussives. His FVC reached a nadir of 2.84 L (54% predicted) on 4 April 1980, increasing over the next month to 4.90 L (90% predicted); the DL_CO_ improved to 31.2 mL/min/mmHg (94% predicted). By the end of April, his weight had returned to baseline and he no longer had a cough.

On 28 April 1980, the patient returned to work in the metal production department, intermittently as a reduction furnace operator. In July and August, he was seen multiple times for rash and skin ulcers on the wrist and hand. In early September, he described a productive cough and his FVC had fallen to 4.63 L (88% predicted). By 11 October 1980, he had lost 7.7 kg and reported progressive cough and exertional dyspnea. His FVC at that time was 4.33 L (83% predicted), with a DL_CO_ of 24.8 mL/min/mmHg (78% predicted). His chest radio graph was normal. A BeLPT drawn on 21 October 1980 was normal (stimulation index of 1.4).

Over the next several months the patient continued to work despite persistent symptoms. Repeat pulmonary function testing on 2 December 1980 showed further declines in FVC, to 3.61 L (68% predicted), and in DL_CO_, to 20.0 mL/min/mmHg (62% predicted). On 2 February 1981, his FVC had fallen further, to 2.83 L (52% predicted). A month later, the patient was put on medical leave and blood was again drawn for BeLPT. That test and a repeat drawn on 17 March 1981 were abnormal (stimulation indices of 9.2 and 11.4, respectively). A chest radiograph that same day was remarkable for a mild diffuse nodular infiltrate (Figure 1B,C). In early April, BAL at the National Institutes of Health (NIH) revealed 47% lymphocytes.

In the ceramics and alloy departments, the patient was likely exposed to insoluble beryllium (e.g., beryllium oxide). In the metal production department, he was likely exposed to soluble (e.g., beryllium fluoride) and insoluble forms of beryllium and other irritants (e.g., ammonium fluoride). The highest TWA GA or BZ samples collected on any given day from the work areas or activities associated with his jobs during his tenure are displayed in Figure 2B, showing that none of the measured air samples exceeded 100 μg/m^3^ and that most were < 10 μg/m^3^. In the reduction furnace, molten beryllium is poured into pots that are carried via a conveyor to a cooling bay. During a 45-min period on 2 March 1980, the patient entered the cooling bay twice to conduct repair work on the conveyor, wearing a negative-pressure half-face respirator. The furnace had just finished pouring, such that the fumes in the cooling bay were considered by the company to be very heavy. The sampling on that shift showed an 8-hr TWA of 5.9 μg/m^3^.

During 3 months away from work (March–June 1981), the patient’s symptoms resolved and his pulmonary function normalized (Figure 2). He returned to work on 15 June 1981, now in the alloy department; he was restricted from working in the furnace area. He remained asymptomatic, but with a persistently elevated lymphocyte count (37–49%) on BAL performed on six occasions from June 1981 to December 1982. A transbronchial biopsy performed at the NIH on 3 January 1983 revealed peribronchial noncaseating granulomas. Fungal stains of the tissue were negative. A follow-up chest radiograph taken in 1997 (Figure 1D) demonstrated reduced lung volumes and a bilateral interstitial infiltrate. He left the plant in 1999 because of CBD.

## Case 2

**Figure 3:**
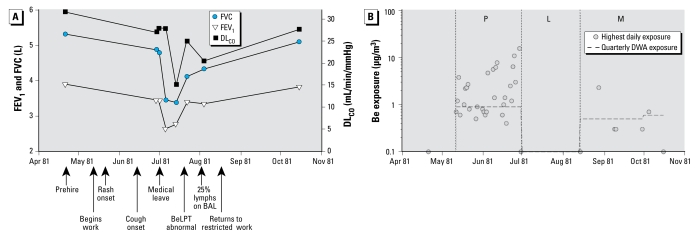
Summary of lung function and beryllium exposure of case 2. (*A*) Results of pulmonary function tests before, during, and after acute work-related illness. (*B*) TWA and quarterly DWA airborne beryllium exposures in patient’s departments during this time period. Abbreviations: L, medical leave; M, maintenance department exposures (beryllium metal, beryllium oxide, copper-beryllium alloy); P, metals production department exposures (beryllium metal, beryllium oxide, beryllium fluoride, ammonium beryllium fluoride, ammonium fluoride, magnesium fluoride).

A healthy 25-year-old male smoker began working at the beryllium production plant on 11 May 1981. His pre employment chest radiograph and pulmonary function tests were normal, including an FVC of 5.33 L (115% predicted) and a DL_CO_ of 31.6 mL/min/mmHg (114% predicted) (Figure 3). He worked in the metal production department operating the fluoride furnace (Kent et al. 2001; Kroschwitz and Howe-Grant 1992; Stefaniak et al. 2003, 2004; White and Burke 1955). On 22 May 1981, he presented to the plant’s medical office with a new rash on the wrists and forearms and was seen multiple times for rash and skin ulcers over the next month. On 16 June 1981, a company physician evaluating him for new onset of recurrent epistaxis noted eroded nasal mucosa bilaterally.

On 26 June 1981 the patient complained of fatigue, shortness of breath, and non-productive cough that had begun 1–2 weeks earlier. Pulmonary function testing demonstrated a fall in FVC, to 4.82 L (104% predicted), and a decrease in DL_CO_, to 27.1 mL/min/mmHg (99% predicted). His chest radiograph was normal. On 1 July 1981, a company physician noted rhonchi (sounds commonly caused by secretions in airways) on chest examination, prescribed a decongestant and an antihistamine, and restricted the patient from further work.

In the metal production department, the patient was likely exposed to soluble and insoluble forms of beryllium and other irritant exposures. Figure 3B displays the highest TWA GA or BZ samples collected on any given day from the work areas or activities associated with his jobs during his tenure, showing that none of the measured air samples exceeded 20 μg/m^3^ and most were < 10 μg/m^3^. Although the fluoride furnaces had a ventilated hood system, fuming sometimes exceeded the ventilation system’s capacity, resulting in the operators calling for an evacuation. Fluoride furnace operators normally remained in the furnace area during evacuations, wearing a negative-pressure half-face respirator. The patient was present for 21 evacuations in his 7 weeks of employment, which was considered by the company to be an unusually high number. The 8-hr continuous GA air samples during these evacuations showed an average beryllium air level of 3.7 μg/m^3^ and a maximum of 15.6 μg/m^3^.

On 7 July 1981, the patient’s FVC had fallen to 3.36 L (72% predicted) and DL_CO_ to 15.7 mL/min/mmHg (57% predicted). A BeLPT drawn that day had a stimulation index of 2.0 (normal range, 1.2–2.0). By 21 July 1981, his FVC had increased to 4.12 L (89% predicted) and DL_CO_ to 25.0 (91% predicted). A repeat BeLPT was abnormal (stimulation index of 5.8). In early August, BAL at the NIH was remarkable for 25% lymphocytes. The skin ulcers were healing, and the cough had resolved.

On 13 August 1981 the patient returned to work at the beryllium plant, restricted from working in the furnace area. A BeLPT drawn 2 days prior was normal (stimulation index of 1.1). His position on return to the plant was in maintenance, where he was likely exposed to less soluble or insoluble forms of beryllium. Pulmonary function subsequently returned to baseline (Figure 3A). He was laid off from work on 16 October 1981. On 12 April 1982, repeat BAL at the NIH had 1% lymphocytes.

In December 1982, the patient had a chest radiograph that showed a rounded soft tissue density in the left lower lung field. BeLPT at that time was normal (stimulation index of 1.3). On 7 February 1983, a chest computed tomography study at the NIH reportedly showed a left-lung nodule, two right-lung nodules, and multiple small, poorly defined parenchymal lesions bilaterally. Open lung biopsy of the left-lung nodule, performed to rule out malignancy, revealed noncaseating granulomas. Fungal and myco-bacterial stains and cultures of the biopsied tissue were negative. The patient returned to work at the beryllium production plant on 26 March 1984 in the laundry and later as a janitor, still restricted from the furnace area. He left the plant in 1992 because of CBD.

## Historical Literature Review

Physicians caring for workers with acute reactions to beryllium in the 1940s described dermatitis, nasopharyngitis, tracheobronchitis, and pneumonitis that developed days to months after initiation of employment (DeNardi et al. 1949; Van Ordstrand et al. 1945). Dermatitis, an eczematous process mainly on exposed skin, affected 25% of new employees and was noted 3–10 days after initial exposure to beryllium salts (DeNardi et al. 1949). Respiratory manifestations appeared on a continuum. Nasopharyngitis was marked by nasal and throat discomfort, mild epistaxis, diffuse mucous membrane edema and hyperemia, and occasional fissuring (Van Ordstrand et al. 1945). It often accompanied tracheobronchitis, which was characterized by nonproductive cough with occasional hemoptysis, and in some cases by low-grade fever, dyspnea, anorexia, and weight loss. Examination of workers with tracheobronchitis revealed early inspiratory rales and decreased vital capacity. Those with tracheo bronchitis who continued to work could develop pneumonitis, the most severe form of the acute reaction. Pneumonitis manifested the symptoms and signs of tracheobronchitis, plus chest pain, severe dyspnea, fatigue, and, in many cases, cyanosis. Although most respiratory disease was insidious in onset, taking several weeks to manifest, a more fulminating form of respiratory disease was recognized after “brief but massive” exposures (DeNardi et al. 1953); in these cases, symptoms developed approximately 72 hr after exposure.

Pneumonitis was distinguished from tracheo bronchitis principally by bilateral changes on chest radiograph that appeared 1–3 weeks after onset of symptoms (DeNardi et al. 1953; Van Ordstrand et al. 1945). Radiographic changes were marked initially by diffuse haziness, followed by the development of irregular areas of infiltration with prominent peribronchial markings, and finally by the resolution of the infiltration and appearance of nodules scattered throughout the lungs (Van Ordstrand et al. 1945). In those who survived, clearing of the chest radiograph occurred over the course of one or more months (Van Ordstrand et al. 1945). Ten percent of those who developed pneumonitis died (DeNardi et al. 1953).

Pathologic review of lung tissue from fatal cases revealed an inflammatory exudate and formation of new connective tissue (Dutra 1948; Freiman and Hardy 1970; Hazard 1959; Machle et al. 1948; Van Ordstrand et al. 1945; Vorwald 1948). Alveoli contained fluid and a predominance of large mononuclear cells, along with moderate numbers of lymphocytes and plasma cells; polymorphonuclear leukocytes were uncommon. Lymphocytes and plasma cells were also found infiltrating the interstitium. In addition to these findings of a nonspecific inflammatory process, Vorwald (1948) described the proliferation of local histiocytes in some cases as evidence of some organization to the inflammation. Dutra (1948) noted that in some cases, clasmatocytes (macrophages) could be seen surrounding fibrinoid material in alveoli, and these cells were sometimes fused to form multinucleated giant cells; others also noted occasional giant cells (Freiman and Hardy 1970). In several cases, Dutra (1948) found septal nodules comprising fibroblasts, lymphocytes, and plasma cells, as well as early granulomas. These findings of an evolution from nonspecific inflammation to granulomatous changes led him to conclude that there was “evidence of transition of the pathologic lesions of the acute condition to those of the chronic one” (Dutra 1948).

The treating physicians in the 1940s attributed acute reactions to exposure to soluble beryllium salts, distinguishing the signs and symptoms they observed from infection by applying the term “chemical” to the disease process. Early impressions were that hypersensitivity played a role in the pathophysiology. The physicians observed that a large proportion of workers with severe dermatitis had a recurrence of skin manifestations when exposed again, despite protective precautions (DeNardi et al. 1949). Furthermore, new workers who manifested dermatitis were more likely to go on to develop respiratory symptoms, such that dermatitis was “considered a rough indicator of individual susceptibility to pulmonary or bronchial irritation” (DeNardi et al. 1949). Indeed, the observations that the dermatitis generally required several days to manifest and that the radiographic changes of the pneumonitis did not appear for a week or more after the onset of symptoms suggested that direct toxic or irritant mechanisms were not solely responsible. For the pneumonitis, the fact that the magnitude of the exposure was not consistently related to the occurrence of disease (in some cases, “no such relation could be established”) was further evidence that “individual sensitivity … undoubtedly … contributed to the occurrence and severity of disease” (DeNardi et al. 1949). Positive results of patch tests with various forms of beryllium in patients with dermatitis (or, in several cases, acute pneumonitis) provided confirmatory evidence of immune system involvement (Curtis 1951, 1959; Shima 1971).

These important early clues to a possible immune mechanism of ABD were dis regarded by later investigators, who used the term “chemical” to refer to a more traditional dose-dependent inhalational intoxication akin to those of “phosgene, nitric oxide, and phosphorus oxychloride” (Sterner and Eisenbud 1951). Despite an acknowledged lack of sufficient data on the “relationship between atmospheric concentrations and incidence of [acute respiratory] disease,” these investigators (Eisenbud et al. 1948) concluded that airborne exposures to beryllium salts at concentrations > 100 μg/m^3^ could produce pneumonitis and that concentrations > 1 mg/m^3^ would consistently do so among all exposed individuals (Eisenbud et al. 1948; Sterner and Eisenbud 1951). They recognized that earlier authors had not been able to establish such an exposure–response relationship (DeNardi et al. 1949; Sterner and Eisenbud 1951). However, they reasoned that cases of ABD that did not conform to these toxicologic parameters reflected the effect of repeated exposures, leading to a cumulation of injury that would lower the threshold for respiratory symptoms (Sterner and Eisenbud 1951). They acknowledged the longer delay between exposure and disease manifestations compared with other causes of acute chemical pneumonitis, but they did not offer an explanation for this discrepancy (Sterner and Eisenbud 1951). In contrast to their concept of ABD’s resulting from a chemical process, they hypothesized that CBD was immune-mediated, postulating the involvement of an antibody response (Sterner and Eisenbud 1951).

## Discussion

The idea of different mechanisms behind beryllium’s acute and chronic effects has been reiterated in the literature and in textbooks in the years since it was first proposed (Balmes 2005; Becklake and Cowie 2000; Churg and Green 1998; Williams 1988). Clinical, epidemiologic, and laboratory-based investigations subsequently have shed much light on the mechanism of CBD. We now know that cellular (rather than humoral) immunity is responsible for its manifestations, that increased susceptibility is conferred through major histocompatibility complex class II genetic variants, and that sensitization to beryllium can be detected via specific lymphocytic proliferative responses measured by the BeLPT (Amicosante and Fontenot 2006; Kreiss et al. 1994; McCanlies et al. 2004). The decline in the occurrence of ABD, however, has limited research into its mechanism. Instead, with only rare suggestions of an immunologic process (Kreiss et al. 2007; Newman and Maier 2003), the conceptualization of ABD as a toxic chemical irritation has remained virtually unchanged. This conceptualization is reflected in descriptions of ABD as indistinguishable from other chemical inhalational injuries, dose-related exposures, and secondary to high-intensity exposures. Such descriptions fail to account for the delayed onset of symptoms and radiographic manifestations, the specific pathologic characteristics of an evolving granulomatous inflammation, and the lack of a clear exposure–response relationship.

The two cases we describe here of acute dermal and respiratory disease that occurred in beryllium metal production workers share many features with the cases of ABD reported in the 1940s. Like those earlier cases, both workers experienced onset of dermatitis about 10 days after starting work in the furnace areas. In case 2, epistaxis and mucosal erosions were evidence of the nasopharyngitis described by the earlier investigators. Both workers subsequently developed respiratory symptoms and marked declines in FVC and DL_CO_, indicating pneumonitis, as observed in the 1940s. Both had remarkable improvement in symptoms and objective findings after removal from the workplace, again consistent with the 1940s experience. In case 1, dermal and respiratory symptoms and pulmonary function abnormalities recurred upon return to the same work environment; in case 2, the worker was reassigned away from the furnace area and did not have a recurrence of acute symptoms. In case 1, delayed radiographic changes during the second, more prolonged episode of acute illness included scattered nodules, a finding also noted late in the course of 1940s cases. Thus, their clinical patterns and occupational exposures, so similar to those described earlier, indicate that these two workers had ABD.

The clinical courses and diagnostic data of these cases provide evidence of a shared immunologic mechanism between ABD and CBD. The timing of the dermatitis suggests a delayed-type hypersensitivity reaction rather than an irritative process, which would be expected to present more rapidly after exposure. Similarly, the progression of respiratory symptoms and signs over weeks or months, accompanied in case 1 by substantial weight loss, is more consistent with an immuno logic process than a chemical insult. The abnormal BeLPT results at the time of acute illness indicate the presence of a cell-mediated immune response to beryllium concurrent with the acute reaction to beryllium. And the lymphocytic predominance seen on BAL fluid analysis at the time of acute illness demonstrates a specific alveolitis, as seen in CBD, rather than the nonspecific inflammatory process of an irritant.

In both cases, lung biopsy performed about 18 months after acute illness, when the workers were asymptomatic, revealed non caseating granulomas, a hallmark of CBD. That ABD can precede and even “merge imperceptibly with” (Freiman and Hardy 1970) CBD has been reported previously (Freiman and Hardy 1970; Hardy 1965; Rees 1979; Tepper et al. 1961). Indeed, Hardy (1951) noted early on that in some cases, CBD could follow ABD “after a variable period of time, with or without further beryllium exposure.” In the survey of beryllium production facility workers (Kreiss et al. 1997), we found that workers who reported a history of cough or breathing problems related to beryllium fluoride had twice the odds of beryllium sensitization or CBD than did workers without that history (Cummings KJ, Kreiss K, unpublished data). Explanations for an association between ABD and subsequent CBD other than a shared immunologic mechanism (e.g., the role of a common relevant exposure) are difficult to refute unequivocally in the absence of experimental data. Yet as the cases presented here demonstrate, the concept of a disease continuum whereby cell-mediated immune recognition of beryllium produces acute symptoms, is active during asymptomatic periods, and is responsible for chronic impairment, is by far the most parsimonious and consistent explanation.

The modern decline in reports of ABD has mirrored the decrease in average exposures. In their 2004 report of new cases, Kim et al. (2004) noted beryllium concentrations ranging from 3 to 112 μg/m^3^, suggesting that affected workers may have had unusually high exposures for the contemporary industrial setting. Yet for the workers in the cases in the present study, the available industrial hygiene data represent the highest TWA exposures for tasks or work areas on a given day associated with the jobs they held. Coupled with the reported use of respirators for some tasks and during upset conditions, the exposure data suggest that these cases of ABD occurred at exposures less than the high concentrations commonly described as necessary to provoke an acute response. This finding is consistent with the lack of a reliable exposure–response relationship noted by the earlier investigators (DeNardi et al. 1949), and with the Japanese experience, where pneumonitis was noted with exposures as low as 25 μg/m^3^ and bronchitis was reported with even lower exposures (Shima 1971).

The present investigation has several limitations. Although the two cases presented here offer a relative wealth of diagnostic information, they do not represent an exhaustive review of all cases. Furthermore, the variations in BeLPT results over time appear to provide a conflicting message about the role of cell-mediated immunity in ABD. Although consistently abnormal results throughout the acute illness may have been more convincing, the test’s specificity (99%) is substantially higher that its sensitivity (66%) (Middleton et al. 2006). Thus, the greater challenge is reconciling the finding of abnormal BeLPT results during acute illness with a chemical mechanism of ABD. Another limitation is that although the available air sampling data were comprehensive and our presentation of the data emphasized highest TWA values, some exposures may have been underestimated, particularly if instantaneous peaks occurred during short- or long-duration sampling and were subject to averaging. Furthermore, we cannot rule out the possibility of unusually elevated airborne concentrations of beryllium that went unmeasured.

Despite these limitations, these cases provide compelling evidence in support of the hypothesis that ABD, like CBD, is an immune-mediated phenomenon. Why some individuals exposed to beryllium manifest ABD only, others CBD only, and still others both ABD and CBD, is likely multi-factorial. Investigators have highlighted the chemical form of beryllium to which a person is exposed. The initial reports of ABD emphasized the role of exposure to soluble forms of beryllium (DeNardi et al. 1949, 1953; Van Ordstrand et al. 1945), and the hypothesis that solubility influenced the manifestation of ABD versus CBD was put forth early on (Machle et al. 1948; Waksman 1959). Solubility facilitates absorption, with salts being more readily dissolved by sweat (Curtis 1951) and respiratory secretions. Furthermore, soluble beryllium, as suggested by Waksman (1959), would be “active in complexing with body constituents,” producing high local antigen concentrations that lead to a vigorous, acute response, whereas insoluble beryllium would be less reactive and remain unaltered for longer periods. Indeed, subsequent research has shown that at the neutral pH in airway epithelial lining fluid, beryllium ions may rapidly complex to form hydroxide (BeOH^+^) and carbonate (BeCO_3_) compounds (Sutton and Burastero 2003) and bind to a range of organic acids and proteins (Scott et al. 2008).

Others have noted variations in persistence of different forms of beryllium in the body. From 10 months to 5 years after recovery from acute pneumonitis related to soluble beryllium salts, follow-up revealed the absence of detectable beryllium in the urine of those patients who had not returned to working with beryllium (DeNardi et al. 1949). In contrast, some workers exposed to insoluble forms of beryllium continued to excrete beryllium 6 years after removal from exposure (DeNardi et al. 1953). More recently, insoluble beryllium metal and oxide have been shown to have dissolution lifetimes of hundreds of days to years in lung airway epithelial lung fluid and alveolar macrophage phagolysosomal fluid (Finch et al. 1988; Stefaniak et al. 2006). Autopsy studies have confirmed that beryllium particles are identifiable in granulomas formed in the lungs of individuals with CBD years after exposure ceased (Butnor et al. 2003; Sawyer et al. 2005; Williams and Wallach 1989). Thus, Stefaniak et al. (2003, 2008) hypothesized that exposure aerosol physical properties, chemical properties, and physicochemical properties control development of beryllium lung burdens, and that the ongoing presence of a lung reservoir of beryllium may be necessary for the development of CBD.

Although early investigation demonstrated that patch testing with poorly soluble beryllium compounds (metal, oxide) and control anions and acids alone failed to produce a reaction (Curtis 1951), it is possible that coincident irritant exposures (e.g., ammonia or hydrofluoric acid from a fluoride furnace or magnesium fluoride from a reduction furnace) play a contributory role in disease manifestation. Investigations of allergic contact dermatitis have found evidence of interplay between irritation and sensitization. Irritant dermatitis can increase the induction of allergic dermatitis, through mechanisms that include skin barrier disruption and cytokine release by non-immune dermal cells (Burkhart 2006; Smith et al. 2002; Zhang and Tinkle 2000). There is evidence for an analogous process in the respiratory tract: irritants such as diesel exhaust (Kleinman et al. 2007; Wichmann 2007) and tobacco smoke (Diaz-Sanchez et al. 2006) can exacerbate responses to known allergens. Thus, it is feasible that coincident irritation of the skin or respiratory tract could promote sensitization and the development of ABD. The role of beryllium’s chemical properties in the development of sensitization and disease remains an area of active inquiry (Day et al. 2005; Stefaniak et al. 2003, 2004, 2006).

The physicians caring for the workers we describe did not initially recognize their patients’ symptoms as those of ABD. Their reluctance to use this diagnosis likely reflected the prevailing notion of ABD as a consequence of particularly high exposures. Publications insisting that ABD had become rare because of improved industrial hygiene were undoubtedly influential (DeNardi 1959; DeNardi et al. 1953; Freiman and Hardy 1970; Williams 1977). Even when beryllium was implicated, an increased risk of CBD was not appreciated. Thus whereas treating physicians in the 1940s recommended avoidance of further beryllium exposure following ABD diagnosis (Van Ordstrand et al. 1945), these two workers continued to be exposed, ultimately in work areas with insoluble beryllium. A better understanding of the pathogenesis of ABD may allow for a more protective approach in the future.

## Conclusions

Initial reports of ABD described dermal and respiratory reactions that were delayed in onset, did not follow clear exposure–response patterns, and often recurred upon reexposure. Pathology showed an evolution from non-specific mononuclear inflammation to more specific granulomatous lesions. This telling evidence was overshadowed by subsequent assertions that ABD represented a dose-dependent chemical injury without pathophysiologic relation to the immune-mediated process of CBD. The cases presented here suggest that rather than two distinct clinical entities, ABD and CBD represent points on a continuum of hypersensitivity reactions to beryllium. Differences in disease presentation and progression are likely influenced by the solubility of the beryllium compound involved and possibly by coexposures. Prudence dictates limitation of further beryllium exposure in both ABD and CBD.
